# Blood Cadmium Level Is a Marker of Cancer Risk in Men

**DOI:** 10.3390/nu16091309

**Published:** 2024-04-27

**Authors:** Róża Derkacz, Wojciech Marciniak, Piotr Baszuk, Monika Wysokińska, Natalia Chrzanowska, Marcin Lener, Tomasz Huzarski, Jacek Gronwald, Tadeusz Dębniak, Cezary Cybulski, Anna Jakubowska, Rodney J. Scott, Jan Lubiński

**Affiliations:** 1 Read-Gene, Grzepnica, ul. Alabastrowa 8, 72-003 Dobra, Poland; roza.derkacz@read-gene.com (R.D.); wojciech.marciniak@read-gene.com (W.M.); 2 Department of Genetics and Pathology, International Hereditary Cancer Center, Pomeranian Medical University in Szczecin, ul. Unii Lubelskiej 1, 71-252 Szczecin, Poland; piotr.baszuk@pum.edu.pl (P.B.); monika.wysokinska@pum.edu.pl (M.W.); natalia.chrzanowska@pum.edu.pl (N.C.); marcin.lener@pum.edu.pl (M.L.); tomasz.huzarski@pum.edu.pl (T.H.); jacek.gronwald@pum.edu.pl (J.G.); tadeusz.debniak@pum.edu.pl (T.D.); cezarycy@pum.edu.pl (C.C.); anna.jakubowska@pum.edu.pl (A.J.); 3 Department of Clinical Genetics and Pathology, University of Zielona Góra, ul. Zyty 28, 65-046 Zielona Góra, Poland; 4 School of Biomedical Sciences and Pharmacy, Faculty of Health, University of Newcastle, Newcastle, NSW 2308, Australia; rodney.scott@newcastle.edu.au; 5 Division of Molecular Medicine, NSW Health Pathology North, Newcastle, NSW 2064, Australia

**Keywords:** cadmium, cancer risk, prospective study

## Abstract

Cadmium (Cd) is a known carcinogen, but its impact on cancer risk at lower concentrations is poorly understood. Previous studies on Cd and cancer risk in men show inconsistent results, prompting further investigation. A prospective cohort study involving 2956 men was conducted. Blood Cd levels were measured, and participants were followed for 78 months to assess cancer incidence. Men with high blood Cd levels (>0.71 µg/L) had a significantly increased risk of cancer compared to those with low levels (<0.19 µg/L) (HR 3.42, *p* < 0.001), particularly among non-smokers (HR 3.74, *p* = 0.003), individuals aged < 60 years (HR 2.79, *p* = 0.017), and ≥60 (HR 4.63, *p* = 0.004). The influence of smoking on cancer risk based on Cd levels was not significant in this study. Blood Cd levels may influence cancer risk in men, emphasizing the importance of minimizing Cd exposure to reduce risk. Confirmation of these results in other populations is essential for effective preventive measures against Cd-related cancers.

## 1. Introduction

Cd and its compounds are commonly known to be carcinogenic in humans. The International Agency for Research on Cancer (IARC) has classified Cd and its compounds as Group 1 carcinogens [1,2], based primarily on its association with a higher risk of lung cancer in cohorts of men occupationally exposed to Cd [3,4]. Some studies have shown the carcinogenic impact of Cd on the development of prostate, kidney, breast, liver, hematopoietic system, urinary bladder, and pancreatic and gastric cancers [5,6,7,8,9]. The majority of past research on the risk of cancer associated with Cd has focused on populations with significant exposure to the element. Little attention has focused on the possible carcinogenic effect of Cd at lower concentrations.

There are 11 prospective studies in the literature evaluating the relationship between dietary Cd intake, whole blood, erythrocytes, urine and nail Cd levels, and cancer risk in men. Statistically significant results have been reported in six studies. Julin et al. observed that a dietary intake of Cd above >20 ng Cd/day increased all cancer risk (RR 1.13, *p* = 0.01) and more specifically, prostate cancer risk (RR 1.29, *p* < 0.01) [9]. Studies correlating male cancer risk with blood Cd levels revealed statistically significant results linking disease with exposure. Cao et al. observed a decreased risk of prostate cancer at low blood Cd levels (OR 0.49, *p* = 0.004) [10]. Deubler et al. reported no association between erythrocyte Cd levels and the risk of B-cell non-Hodgkin lymphoma, but a strong inverse association in patients with multiple myeloma. These results were significant in the entire cohort that included both women and menhowever, the results were insignificant when only male data were analyzed [11]. Duell et al. showed an increasing trend in the risk of pancreatic cancer with an increasing level of Cd in erythrocytes (OR 1.87, p-trend = 0.04) [12]. Additionally, Park et al. observed that urine Cd level ≥ 1.96 µg/L correlated with an increased cancer risk of any cancer (HR 1.41, *p* = 0.03) [13]. Cigan et al. recently investigated the correlation between urinary cadmium levels and the likelihood of lung cancer in current smokers [14]. They observed that urinary Cd levels were positively associated with lung cancer risk (HR 1.48, *p* = 0.0002).

In other prospective studies, a statistically significant correlation between Cd levels and cancer risk was not observed [15,16,17,18,19]. Together, the data reported to date indicate there is no consistency in the association of Cd with cancer risk. Therefore, we extended studies of Cd exposure and cancer in men by examining the concentration of blood Cd and correlating this with cancer incidence.

## 2. Materials and Methods

### 2.1. Study Subjects

Men were invited to participate in the study between 2011 and 2018. All participants were patients of outpatient clinics at the Hereditary Cancer Center in Szczecin. Most patients had a positive family history of cancer. The prospective cohort consisted of 2956 male volunteers unaffected by cancer at the time of recruitment (date of the blood draw). All participants provided written informed consent to participate in the study and agreed to supply a blood sample for research purposes. From each patient, information about the smoking status (yes, no) and family history of cancer were collected (Table 1). Smoking status was classified as *yes* when the patient was currently smoking or *no* if the participant had not smoked during the past 10 years. The study was conducted in accordance with the Declaration of Helsinki. All participants signed an informed consent document before donating a blood sample.

During the 78 months of the follow-up, 144 new cancers were diagnosed. Data regarding incident cancers were obtained from the medical and pathology records of the treating hospitals. Table 2 presents cancer’s location in the study group—40% of cancers were located in the prostate.

### 2.2. Laboratory Analysis

Participants provided a 10 mL blood sample at recruitment. An aliquot of 1.5 mL of whole blood was taken and stored at −80 °C. Phlebotomy occurred between 8 a.m. and 12 p.m. from Monday to Friday. The patients fasted for four hours prior to venipuncture.

Total blood Cd concentration was measured by the inductively coupled plasma mass spectroscopy (ICP-MS) technique using an Elan DRC-e (PerkinElmer, Waltham, MA, USA) instrument [20]. Cd was measured in DRC mode with oxygen (O_2_, purity > 0.9999) as a reaction gas for removing spectral interference [21]. Rhodium was chosen as an internal standard to compensate for instrument drift and matrix effects. All the parameters of Elan DRC-e used during measurement are available on request.

The blank reagent was composed of high-purity water (>18 MΩ), TMAH (AlfaAesar, Kandel, Germany), Triton X-100 (PerkinElmer, Shelton, CT, USA), n-butanol (Merck, Darmstadt, Germany), and disodium EDTA (Sigma Aldrich, Steinheim, Germany). The matrix-matched technique was used for calibration to ensure optimal accuracy. The calibration curve standards (0.1; 0.2; 0.5 µg/L) were created by diluting a blank reagent with a stock solution (50 µg/L) of 10 mg/L Multi-element Calibration Standard 3 (PerkinElmer Pure Plus, PerkinElmer, Shelton, CT, USA). The linear coefficient consistently exceeded 0.999. The accuracy and precision of the method were verified using three different certified reference materials—NIST 955c (NIST, Gaithersburg, MD, USA), Plasmonorm Whole Blood Level 1 (Clincheck, Berlin, Germany), and BCR 634 (Sigma Aldrich).

### 2.3. Statistics

Information on incident cancers was retrieved from the medical and pathology records of the treating hospitals. Men were followed from the date of blood draw to the first diagnosis of cancer, death from another cause, or the date of the last follow-up.

Quartiles of blood Cd levels were estimated based on the distribution in all unaffected men. Univariable and multivariable hazard ratios were generated using the Cox proportional hazard model for Cd by quartile (using the first (lowest) quartile as the reference). Multivariable hazard ratios were adjusted for age and smoking status (yes, no). The dataset was then analyzed for subgroups defined by age (<60, ≥60 years old) and smoking status (yes, no). All statistical analyses were conducted using R version 4.0.3.

## 3. Results

The study group consisted of 2956 men who were followed for 78 months from the date of blood draw. During the follow-up period, 144 new cancers were diagnosed. The average age of the study subjects at enrollment was 53 years (range 33–87 years). Blood Cd levels were higher in smokers (1.18 µg/L) compared to non-smokers (0.28 µg/L).

Men with high Cd concentration in blood (>0.71 µg/L) had a more than three-fold increased risk of cancer compared to men with low Cd concentration (<0.19 µg/L) (HR 3.42; 95%CI: 1.67–7.01; *p* < 0.001) (Table 3). Because of differences between blood Cd levels among smokers and non-smokers, we divided the cohort by smoking status. Non-smokers had a decreased risk of cancer if they had low blood Cd levels (Table 4). However, no statistically significant results were observed for smokers (Table 5). Additionally, we divided the cohort by age: <60 and ≥60 years old. We observed significant results in both groups (Table 6 and Table 7).

## 4. Discussion

Exposure to Cd and its compounds and their negative impact on the body is the basis of many research studies. However, we found only 11 prospective studies evaluating the correlation between Cd exposure and cancer risk in men. All prospective studies are presented in Appendix A. As discussed in the introduction, the results of published reports do not provide any definite conclusions about Cd exposure and cancer risk.

In the current study, the results are statistically significant for the entire cohort, even without accounting for smoking status. On subgroup analysis, the results reveal that men who have never smoked but have higher levels of blood Cd are at an increased risk of developing cancer. Men who smoked (average rate, 22.3 pack years per subject) were independently examined in this study. When we compared the levels of Cd in the lowest quartile of smokers, there was no difference in the cancer rate between quartile 1 (reference level) and the other 3 quartiles. It is generally accepted that smokers have higher Cd levels [22,23,24] and smoking is associated with cancer risk [25]. Nevertheless, among smokers, we did not see any increased risk of cancer depending on blood Cd levels.

In this study, participants were patients of the Hereditary Cancer Centre in Szczecin; thus, the majority had a positive family history of cancer. According to the GLOBOCAN 2022 report [26], the most common cancers among men are prostate (20.6%), lung (17.6%), and colorectal cancer (14.3%). However, in this study group, prostate cancer accounted for the highest percentage (40%), followed by colorectal cancer (13%) and kidney cancer (13%). Patients with lung cancer represented only 4% of all cases. The significant difference between the population frequency of lung cancer and its frequency in the presented study group may contribute to the absence of a correlation between blood Cd levels and cancer in smokers. In a separate prospective study, Cigan et al. analyzed urine and showed that Cd levels were strongly associated with lung cancer risk among smokers [14].

Smoking is one of the main sources of high-dose Cd exposure. A single cigarette contains approximately 1–2 µg of Cd, of which about 10% is inhaled. With an absorption lung rate of 40–50%, an individual smoking 20 cigarettes daily may absorb approximately 1 µg of Cd [27,28]. For non-smokers and individuals not occupationally exposed to Cd compounds, groceries constitute the primary source of Cd [29,30,31]. Krajcovicoya-Kudlackova et al. [32,33] reported that vegans and vegetarians have significantly higher blood Cd concentrations compared to people with a mixed diet, with values of 3.15 ± 0.77, 1.75 ± 0.37, and 0.45 ± 0.04 µg Cd/L, respectively. Vegetarians typically consume larger quantities of vegetables, fruits, and grains, leading to an elevated intake of Cd. Additionally, soy products are a common substitute for animal products used by Vegans/Vegetarians, and contain 7.6 ± 0.1 µg Cd/kg [34]—this is not the richest source of Cd, but it is one of the main ingredients of vegetarian and vegan cuisine.

Additionally, many factors could affect Cd absorption, such as age, sex, chemical form of Cd, dosage [35], and interaction with other elements, such as copper, iron, zinc, calcium, and magnesium [28,36,37]. Moreover, the type of food product is not without significance. The highest concentration of Cd is in seafood (selfish, scallops, mussels) and offal (kidneys, liver). In plants, the highest capacity for accumulation is predominantly located in leaves and roots. Vegetable products with high Cd content include spinach, lettuce, cereals, potatoes, oilseeds, soy, and cocoa-based products [29,30,31].

There are several limitations of the study. We collected only information about smoking history and family history of cancer. We do not have information about other risk factors: passive smoking, occupational exposure, body mass index, diet, alcohol consumption, and others. Validating our findings through confirmation by other researchers is crucial to ensure the reliability and generalizability of our results regarding Cd exposure. This study is part of an ongoing program to study a panel of micronutrients and heavy metals as possible risk factors for cancer in high-risk men.

## 5. Conclusions

In conclusion, our data suggest that blood Cd levels may influence cancer risk in men. If confirmation of the results presented herein by other research groups occurs, we should focus on minimizing Cd exposure with the goal of reducing the risk of cancer.

## 6. Patents

Work reported in this manuscript has been submitted to the Patent Office of the Republic of Poland. The patent was accepted in 2023 and received the number Pat. 243863.

## Figures and Tables

**Table 1:** Characteristics of men included in the study.

	All Cohort *N* = 2956	Unaffected *N* = 2812	New Cancer Diagnosis *N* = 144	Mean Cd Level, µg/L (Range)
**Age**				
range	33–87	33–87	36–76	-
mean	53	53	60.5	-
**Follow-up (months)**				
range	6–120	29–120	6–103	-
mean	76	78	48	-
**Smoking**				
no	1743 (59%)	1656 (56%)	87 (3%)	0.28 (0.02–2.34)
yes	1213 (41%)	1156 (39%)	57 (2%)	1.18 (0.08–11.82)

**Table 2:** Cancer location in study group.

Cancer Site	*N* (%)
prostate	58 (40%)
colon	13 (9%)
kidney	13 (9%)
bladder	12 (8%)
melanoma	12 (8%)
circulatory system	7 (5%)
lung	6 (4%)
liver	4 (3%)
head, neck, brain	4 (3%)
thyroid	4 (3%)
lymphatic system	2 (1.5%)
pancreatic	2 (1.5%)
skin	2 (1.5%)
stomach	2 (1.5%)
breast	1 (0.7%)
esophagus	1 (0.7%)
testis	1 (0.7%)

**Table 3:** Association Between Blood Cd Level and Cancer Risk. Entire Cohort (*n* = 2812).

			Univariable COX Regression			Multivariable COX Regression ^C^		
Blood Cd Level, Quartiles	Unaffected, *N* = 2812 ^A^	New Cancer Diagnosis, *N* = 144 ^A^	HR ^B^	95% CI ^B^	*p*-Value	HR ^B^	95% CI ^B^	*p*-Value
0.02–0.19	678 (24%)	14 (9.7%)	1.0	—	—	1.0	—	—
0.20–0.32	727 (26%)	39 (27%)	2.46	1.33, 4.52	0.004 *	2.15	1.17–4.0	0.014 *
0.33–0.70	698 (25%)	45 (31%)	2.94	1.62, 5.36	<0.001 *	2.65	1.42–4.92	0.002 *
0.71–11.82	709 (25%)	46 (32%)	2.84	1.56, 5.17	<0.001 *	3.42	1.67–7.01	<0.001 *

^A^ *n* (%). ^B^ HR = Hazard Ratio, CI = Confidence Interval. ^C^ Risk factors: age (<60, ≥60 y.o.), smoking (yes, no). The symbol * denotes a statistically significant result (*p*-value < 0.05).

**Table 4:** Association Between Blood Cd Level and Cancer Risk. Subgroup—non-smoking men (*n* = 1656).

			Univariable COX Regression			Multivariable COX Regression ^C^		
Blood Cd Level, Quartiles	Unaffected, *N* = 1656 ^A^	New Cancer Diagnosis, *N* = 87 ^A^	HR^B^	95% CI ^B^	*p*-Value	HR ^B^	95% CI ^B^	*p*-Value
0.02–0.15	388 (23%)	6 (6.9%)	1.0	—	—	1.0	—	—
0.16–0.22	421 (25%)	17 (20%)	2.60	1.02, 6.59	0.044 *	2.26	0.89, 5.75	0.086
0.23–0.32	420 (25%)	24 (28%)	3.50	1.43, 8.55	0.006 *	2.68	1.09, 6.61	0.032 *
0.33–2.34	427 (26%)	40 (46%)	5.63	2.39, 13.3	<0.001 *	3.74	1.56, 8.95	0.003 *

^A^ *n* (%). ^B^ HR = Hazard Ratio, CI = Confidence Interval. ^C^ Risk factors: age (<60, ≥60 y.o.). The symbol * denotes a statistically significant result (*p*-value < 0.05).

**Table 5:** Association Between Blood Cd Level and Cancer Risk. Subgroup—smoking men (*n* = 1156).

			Univariable COX Regression			Multivariable COX Regression ^C^		
Blood Cd Level, Quartiles	Unaffected, *N* = 1156 ^A^	New Cancer Diagnosis, *N* = 57 ^A^	HR ^B^	95% CI ^B^	*p*-Value	HR ^B^	95% CI ^B^	*p*-Value
0.08–0.41	284 (25%)	9 (16%)	1.0	—	—	1.0	—	—
0.42–0.83	294 (25%)	13 (23%)	1.29	0.55, 3.03	0.6	1.12	0.48, 2.62	0.8
0.84–1.42	288 (25%)	18 (32%)	1.79	0.80, 3.99	0.2	1.53	0.68, 3.43	0.3
1.43–11.82	290 (25%)	17 (30%)	1.70	0.76, 3.81	0.2	1.49	0.66, 3.35	0.3

^A^ *n* (%). ^B^ HR = Hazard Ratio, CI = Confidence Interval. ^C^ Risk factors: age (<60, ≥60 y.o.).

**Table 6:** Association Between Blood Cd Level and Cancer Risk. Subgroup—<60 years old (*n* = 1990).

			Univariable COX Regression			Multivariable COX Regression ^C^		
Blood Cd Level, Quartiles	Unaffected, *N* = 1990 ^A^	New Cancer Diagnosis, *N* = 58 ^A^	HR ^B^	95% CI ^B^	*p*-Value	HR ^B^	95% CI ^B^	*p*-Value
0.02–0.19	558 (28%)	9 (16%)	1.0	—	—	1.0	—	—
0.20–0.32	512 (26%)	14 (24%)	1.61	0.70, 3.73	0.3	1.67	0.72, 3.88	0.2
0.33–0.70	439 (22%)	19 (33%)	2.53	1.14, 5.58	0.022 *	2.79	1.20, 6.48	0.017 *
0.71–11.82	481 (24%)	16 (28%)	1.90	0.84, 4.30	0.12	2.35	0.84, 6.58	0.10

^A^ *n* (%). ^B^ HR = Hazard Ratio, CI = Confidence Interval. ^C^ Risk factors: smoking (yes, no). * statistically significant result (*p*-value < 0.05).

**Table 7:** Association Between Blood Cd Level and Cancer Risk. Subgroup—≥60 years old (*n* = 822).

			Univariable COX Regression			Multivariable COX Regression ^C^		
Blood Cd Level, Quartiles	Unaffected, *N* = 822 ^A^	New Cancer Diagnosis, *N* = 86 ^A^	HR ^B^	95% CI ^B^	*p*-Value	HR ^B^	95% CI ^B^	*p*-Value
0.04–0.19	120 (15%)	5 (5.8%)	1.0	—	—	1.0	—	—
0.20–0.32	215 (26%)	25 (29%)	2.58	0.99, 6.75	0.053	2.69	1.03, 7.04	0.043 *
0.33–0.70	259 (32%)	26 (30%)	2.28	0.87, 5.93	0.092	2.69	1.02, 7.05	0.044 *
0.71–10.89	228 (28%)	30 (35%)	2.74	1.06, 7.06	0.037 *	4.63	1.62, 13.2	0.004 *

^A^ *n* (%). ^B^ HR = Hazard Ratio, CI = Confidence Interval. ^C^ Risk factors: smoking (yes, no). The symbol * denotes a statistically significant result (*p*-value < 0.05).

## Data Availability

The datasets used and/or analyzed during the current study are available from the corresponding author on reasonable request due to legal statements.

## Appendix A

**Table A1:** Characteristics of prospective studies assessing the risk of cancer depending on the concentration of Cd in the diet, urine and nails.

Cancer	Population	Sex, Age (Range, Mean)	Follow-Up	Risk Factors	N, New Cancer Diagnosis	Cd Concentration	Results *	Literature
***Whole blood Cd concentration***								
Breast Ovarian Prostate Testicular	American	women, men >20	19 years	age, education, race/ethnicity, poverty income ratio, BMI, marital status	*N* = 94,337 (1718) Breast—788 Ovarian—113 Prostate—784 Testicular—33	quantile (mean) [µg/L] 0.25|0.17 0.50|0.30 0.75|0.61	**prostate** OR 0.49 (0.30–0.80) *p* = 0.004 **testicular** OR 0.54 (0.06–4.55) *p* = 0.57	Cao et al., 2023 [10]
Skin (non-melanoma)	American	women, men >20 (48.2)	7 years	age, sex, ethnicity, education, marital status, poverty income ratio, alcohol drinking status, smoking status, BMI, systolic blood pressure, creatinine, physical activity (MET score), diabetes, hypertension, hyperlipidemia	*N* = 16,034 (202)	quartiles [-] 1. 0.62–1.69 2. 1.78–2.76 3. 2.85–4.98 4. 5.07–115.93	*Quartile 1 vs. 4* OR 0.87 (0.7–1.09) p-trend = 0.151	Wang and Yu., 2023 [15]
***Erythrocytes Cd concentration***								
B-cell non Hodgkin lymphoma and multiple myeloma	American CPS-II NC	women, men (40–90)	(ongoing since 1992)	Case-control matched by race, age, sex, blood draw date. smoking status, alcohol use, gender, age at diagnosis, time between blood draw and diagnosis, and age at blood draw were also completed	*N* = 1125 (375)	quartiles [µg/L] **total** 1. <0.40 2. 0.40–<0.56 3. 0.56–<0.77 4. >0.77 **men** 1. <0.36 2. 0.36–<0.49 3. 0.49–<0.70 4. >0.70	*continuously (per 1 SD increase)* *Multiple myeloma* **total** RR 0.59 (0.38–0.89) **men** RR 0.64 (0.38–1.07)	Deubler et al., 2020 [11]
B-cell non Hodgkin lymphoma and multiple myeloma	Italian EPIC-Italy Swedish NSHDS	EPIC-Italy women, men (35–70) NSHDS women, men (40–60)	2–16 years	Case-control matched by population, sex, age (±5 years), centre, date of blood collection (±6 months) sex, age, centre, batch and sample date	EPIC-Italy *N* = 168 (84) NSHDS *N* = 372 (186)	quartiles [µg/L] **total** 1. 0.14–0.32 2. 0.32–0.50 3. 0.50–0.74 4. 0.74–5.22 **men** 1. 0.14–0.26 2. 0.26–0.37 3. 0.38–0.80 4. 0.80–5.22	*quartile 1 vs. 4* B-cell NHL **total** OR 1.09 (0.61–1.93) *p* = 0.78 **men** OR 0.65 (0.27–1.56) *p* = 0.33 Multiple myeloma **total** OR 1.16 (0.40–3.40) *p* = 0.79 **men** OR 0.84 (0.11–6.62) *p* = 0.50	Kelly et al., 2013 [16]
Pancreatic	(not reported) EPIC cohort	women, men (age not reported)	12.2 years	Case-control matched by age, sex, study center Smoking, alcohol, BMI, diabetes, education and other metals	*N* = 1331 (429)	quantiles not reported	OR_log2Cd_1.13 (1.01–1.27) OR_Q5v1_1.87 (1.13–3.08) p-trend = 0.04	Duell et al., 2018 (abstract) [12]
***Dietary Cd intake (FFQ questionnaire)***								
Prostate	Danish	men (50–65)	13 years	educational level (<8 yrs, 8–10 yrs, >10 yrs). Smoking (never, former, current), BMI, waist-to-hip ratio, physical activity (MET score)	*N* = 27,178 (1567)	tertiles [ngCd/day] 1. <14 2. 14–18 3. >18	*tertile 1 vs. 3* IRR 0.97 (0.86–1.10)	Eriksen et al., 2015 [17]
Prostate + all cancer sites	Swedish	men (45–79)	10.8 years	age (years), family history of prostate cancer (yes, no, unknown), years of education (<12, ≥12 years), BMI (18.5–<25, 25–<30 and ≥30), waist circumference (<94, 94–102 and ≥102 cm), metabolic equivalent (MET) hours per day (quartiles), smoking status (ever, never), total energy intake (kcal), alcohol consumption (<0.1, 0.1–<5, 5–<10, 10–<15 and ≥15 g/day), selenium, lycopene and calcium (mg/day, tertiles)	*N* = 41,089 (3085) **prostate cancer** 894 (794 advanced and 326 fatal)	tertiles [ngCd/day] 1. <17 2. 17–20 3. >20	*tertile 1 vs. 3* **total** RR 1.13 (1.03–1.24) *p* = 0.01 **prostate cancer** RR 1.29 (1.08–1.53) *p* < 0.01 **advanced prostate cancer** RR 1.05 (0.87–1.25) *p* = 0.7 **fatal prostate cancer** RR 1.14 (0.86–1.51) *p* = 0.35	Julin et al., 2012 [9]
all cancer sites	Japanese	women, men (45–47)	9 years	age, living area, BMI, smoking history, frequency of alcohol consumption, physical activity, consumption of meat, soy, vegetables, fruit, menopause status (yes, no), use of exogenous hormones in women	*N* = 90,383 (5849)	quartiles (median) [ngCd/day] **men** 1. 18.4 2. 24.3 3. 29.3 4. 37.5	*quartile 1 vs. 4* **men** HR 0.94 (0.82–1.08) *p* = 0.46	Sawada et al., 2012 [18]
***Urine Cd concentration***								
Thyroid + all cancer sites	South Korean	women, men, ≥19	8 years	age, sex, region (random effect), enrollment year (random effect), education achievement, smoking status, and job status	*N* = 5406 (371) women 2004 men 3402 **all cancers** women—166 men—137 **thyroid cancer** women—60 men—8	tertiles [µg/g creatinine] 1. <0.91 2. 0.91–1.96 3. ≥1.96	*tertile 1 vs. 3* **total** HR 1.41 (1.01–1.95) *p* = 0.03 **thyroid cancer** HR 2.28 (0.93–3.91) *p* = 0.03	Park et al., 2021 [13]
Lung	Five ethnicity (Africans Americans, Native Hawaiians, Whites, Latinos, Japanese Amercians)	women, men, ≥45	13.4 years	Only current smokers age, sex (men/women), race (ethnicity African American, Native Hawaiian, White, Latino, Japanese American), BMI, creatinine (mg/dL; log).	*N* = 2309 (140) women—1241 (63) men—1068 (77) Adenocarcinoma—42 Squamous cell carcinoma—38 Small cell lung cancer—21 Unspecified—22	geometric mean (GM)	*Cd urine level vs. lung cancer* *Model 1* HR 1.48 (1.21–1.82) *p* = 0.0002 *Adenocarcinoma* HR 1.75 (1.25–2.46) *p* = 0.001 *Squamous cell carcinoma* HR 0.96 (0.62–1.49) *p* = 0.87 *Small cell lung cancer* HR 1.54 (0.92–2.57) *p* = 0.101 *Unspecified* HR 1.64 (1.05–2.56) *p* = 0.030	Cigan et al., 2023 [14]
***Toenail Cd concentration***								
Prostate	American	men (58–74)	not reported	Cases and controls were matched on age (eligible non-cases nearest in age, with one control being older and one younger), race, date of blood collection (typically within 2 weeks), and size of toenail clipping (small, medium, large) Risk factors were not taken into account.	*N* = 342 (115)	quintiles [ppb] 1. 10.8 2. 28.7 3. 54.5 4. 104.4 5. 310.8	*quintile 1 vs. 5* OR 0.70 (0.36–1.37) *p* = 0.9	Platz et al., 2002 [19]

* In all studies, the lowest Cd concentration was used as a reference. CPS-II NC—Cancer Prevention Study II Nutrition Cohort. NSHDS—Northern Sweden Health and Disease Study. EPIC—European Prospective Investigation into Cancer and Nutrition.
